# The expression of TRMT2A, a novel cell cycle regulated protein, identifies a subset of breast cancer patients with *HER2 *over-expression that are at an increased risk of recurrence

**DOI:** 10.1186/1471-2407-10-108

**Published:** 2010-03-22

**Authors:** David G Hicks, Bagi R Janarthanan, Ramya Vardarajan, Swati A Kulkarni, Thaer Khoury, Daniel Dim, G Thomas Budd, Brian J Yoder, Raymond Tubbs, Marshall T Schreeder, Noel C Estopinal, Rodney A Beck, Yanling Wang, Brian Z Ring, Robert S Seitz, Douglas T Ross

**Affiliations:** 1 University of Rochester, Rochester, New York, USA; 2 The Cleveland Clinic Foundation, Cleveland, Ohio, USA; 3 Clearview Cancer Center, Huntsville, Alabama, USA; 4 Clarient Inc, Huntsville, Alabama, USA; 5 Clarient Inc, Aliso Veijo, California, USA

## Abstract

**Background:**

Over-expression of *HER2 *in a subset of breast cancers (*HER2*+) is associated with high histological grade and aggressive clinical course. Despite these distinctive features, the differences in response of *HER2*+ patients to both adjuvant cytotoxic chemotherapy and targeted therapy (e.g. trastuzumab) suggests that unrecognized biologic and clinical diversity is confounding treatment strategies. Furthermore, the small but established risk of cardiac morbidity with trastuzumab therapy compels efforts towards the identification of biomarkers that might help stratify patients.

**Methods:**

A single institution tissue array cohort assembled at the Clearview Cancer Institute of Huntsville (CCIH) was screened by immunohistochemistry staining using a large number of novel and commercially available antibodies to identify those with a univariate association with clinical outcome in *HER2*+ patients. Staining with antibody directed at TRMT2A was found to be strongly associated with outcome in *HER2*+ patients. This association with outcome was tested in two independent validation cohorts; an existing staining dataset derived from tissue assembled at the Cleveland Clinic Foundation (CCF), and in a new retrospective study performed by staining archived paraffin blocks available at the Roswell Park Cancer Institute (RPCI).

**Results:**

TRMT2A staining showed a strong correlation with likelihood of recurrence at five years in 67 *HER2*+ patients from the CCIH discovery cohort (HR 7.0; 95% CI 2.4 to 20.1, p < 0.0004). This association with outcome was confirmed using 75 *HER2*+ patients from the CCF cohort (HR 3.6; 95% CI 1.3 to 10.2, p < 0.02) and 64 patients from the RPCI cohort (HR 3.4; 95% CI 1.3-8.9, p < 0.02). In bivariable analysis the association with outcome was independent of grade, tumor size, nodal status and the administration of conventional adjuvant chemotherapy in the CCIH and RPCI cohorts.

**Conclusions:**

Studies from three independent single institution cohorts support TRMT2A protein expression as a biomarker of increased risk of recurrence in *HER2+ *breast cancer patients. These results suggest that TRMT2A expression should be further studied in the clinical trial setting to explore its predictive power for response to adjuvant cytotoxic chemotherapy in combination with *HER2 *targeted therapy.

## Background

A major challenge in the treatment of breast cancer is to accurately identify those patients who are more likely to develop recurrence so that appropriate therapy can be selected. Significant advances have been made in the development of combination chemotherapy regimens along with the development of effective targeted therapeutics[1]. However, the selection of patients who are likely to benefit from such treatment remains challenging and has necessitated a search for new molecular biomarkers which could help to better predict the likelihood of recurrence as well as the potential benefit from such adjuvant treatment approaches.

The HER2 gene is amplified in 15-20% of breast cancers and this molecular alteration carries with it a more aggressive clinical course [2-4]. A *HER2 *targeted monoclonal antibody, Trastuzumab, has been evaluated in four large randomized clinical trials demonstrating significant benefits in disease free survival (DFS) with the addition of one year of Trastuzumab to adjuvant chemotherapy[5,6]. However there appears to be clinical heterogeneity in the response to Trastuzamab with a significant number of patients demonstrating either de novo or acquired resistance[6,7]. In addition, a risk of cardiotoxicity has been identified in patients treated with adjuvant Trastuzumab, especially following adjuvant anthracyclines, which may be irreversible in small fraction of patients [8,9]. Given the small but consequential risk of cardiac morbidity associated with Trastuzumab treatment, even in patients with normal baseline cardiac function, there could be a clinical benefit to identifying early stage patients at relatively high risk of recurrence in order to better weigh the risk versus benefit of Trastuzumab treatment.

The clinical heterogeneity of *HER2 *positive tumors is in part reflected in biologic heterogeneity as assessed by gene expression profiling [10-12]. *HER2 *positive tumors identified with immunohistochemistry and/or fluorescence in situ hybridization exhibit variation in global gene expression patterns related in part to expression of hormone receptor related genes and/or signatures that distinguish the aggressive luminal B tumor subtype[13]. Estrogen receptor expression has been investigated as a clinically useful classifier for *HER2 *positive patients but has shown inconsistent results[14]. However, the hormone receptor status of breast cancer does not appear to influence the likelihood of clinical benefit from trastuzumab therapy for *HER2 *positive tumors[5,15].

We have endeavored to translate gene expression based classification of carcinoma into IHC reagents that can be used to discover and validate the relationship between tumor classification and clinically significant phenotypes[16]. In the current study, we sought to investigate candidate IHC markers that could better define *HER2 *biologic diversity and or stratify *HER2*+ breast cancer into significantly different prognostic categories. We previously screened a large number of novel commercially available antisera, targeted by gene expression data, to identify panels of antibodies useful for breast tumor classification[16]. In this study, we queried this dataset for biomarkers associated with outcome in *HER2 *expressing tumors and identified TRMT2A (previously known as HTF9C), a novel cell cycle regulated protein, as associated with aggressive clinical course [16,17]. We confirm this association in two independent clinical cohorts. The identification of a biomarker that reproducibly stratifies *HER2 *positive breast cancer patients into groups with different clinical outcomes may aid clinical decision-making. Furthermore, TRMT2A is a strong candidate biomarker worthy of further investigation as a predictive marker of response to adjuvant chemotherapy and *HER2 *targeted therapy.

## Methods

### Patient samples

Single institutional breast cancer cohorts from the Clearview Cancer Institute of Huntsville (CCIH), Cleveland Clinic Foundation (CCF), and Roswell Park Cancer Institute (RPCI) were used in this study. In all cohorts, patient tumor paraffin blocks were assigned an anonymous unique identifier linked to a clinical data base that contained treatment and outcome data blinded to investigators in this study. IRB approval was obtained for the use of patient blocks at each respective institute. Patients were excluded for study if they were male, had metastatic or multicentric disease at time of diagnosis, or had received trastuzumab in an adjuvant setting. Some study patients were treated variably with adjuvant cytotoxic chemotherapy. 67 *HER2*+ and 75 *HER2*+ cases were identified by IHC staining in the CCIH and CCF tissue arrays. The RPCI cohort was assembled by retrospective review of the pathology archives from January 1996 to January, 2006 to identify *HER2*+ breast cancer patients who where 3+ positive by immunohistochemistry using the Herceptest™ test kit (Dako, Carpenteria Ca). HER2 IHC status for all sixty-four cases identified was confirmed by re-staining. The clinicopathologic characteristics for the three patient cohorts are summarized in table 1. For the purpose of generating clinical datasets, patients were censored at the time of contralateral breast cancer diagnosis, diagnosis of a new primary, or at the time of their last visit for patients clinically evaluated as disease free.

**Table 1:** Clinical and Pathologic Features of Study Patients

		CCIH		CCF		RPCI
		Total	Her2+	Total	Her2+	Her2+

ER	N	452	69	240	81	64

	+	311	38	186	59	32

	-	135	31	42	20	28

	n/a	6	0	12	2	4

PR	+	234	20	128	30	10

	-	204	48	104	48	46

	n/a	14	1	8	3	8

Age	Mean	58	56	62	61	56

	<50	132	25	57	25	23

	> = 50	320	44	183	56	41

Nodal Stage	0	259	34	135	52	23
	
	1	176	32	56	16	31

	2	8	3	24	10	5

	3	0	0	8	2	3

	n/a	9	0	17	1	2

Tumor Stage	1	234	26	133	42	29
	
	2	168	35	83	29	22

	3	21	2	12	4	5

	4	12	4	10	5	5

	n/a	17	2	2	1	3

Clinical Stage	1	172	19	90	31	15
	
	2A	133	19	62	27	23

	2B	92	21	26	7	11

	3A	15	1	27	9	7

	3B	16	7	8	4	3

	3C	0	0	8	2	3

	n/a	24	2	19	1	2

Grade	1	69	5	31	7	4

	2	175	21	101	35	22

	3	162	32	72	34	35

	n/a	90	11	36	5	3

Chemo	None	250	28	115	31	18

	Adjuvant	241	41	74	22	45

	n/a	5	0	51	28	1

### Tissue arrays and immunohistochemistry

Each tissue microarray (TMA) block contained a single 0.6 mm core sampled from representative paraffin blocks from each patient. Tissue array sections were de-paraffinized and dehydrated by submerging in xylene three times for 10 minutes each, followed by rinsing three times in 100% ethanol, two times in 95% ethanol and then treated by microwaving boiling for 11 minutes in 10 uM buffered citrate pH 6.0. Slides were allowed to cool to room temperature, and rinsed in distilled water followed by PBS. Slides were dipped in 0.03% hydrogen peroxide followed by rinsing with PBS and then stained using dilutions of antibodies in DAKO Diluent (DAKO Cytomation Inc.) for one hour at room temperature. Initial studies were performed with rabbit polyclonal affinity purified antibodies targeting a peptide located in the amino terminal third of the protein (s0545). A novel rabbit monoclonal antibody (S0722) directed to the same peptide was generated which showed very similar staining pattern to the polyclonal antibody as judged by comparison across a large breast cancer tissue array cohort (data not shown). The RPCI staining was performed using the rabbit monoclonal antibody. For each antibody, dilutions were first tested on a small 'titer' tissue array that had breast cancer cases with positive and negative cases in addition to a set of tumor derived cell lines suspended in paraffin. Secondary antibody was applied for one hour, and staining visualized using the DAKO Cytomation Envision staining kit according to the manufacturer's instructions. Cases were considered TRMT2A expressing if greater than 10% of cells showed cytoplasmic staining. Most positive cases show uniform staining across tumor cells within the sample. Nuclear staining in the absence of cytoplasmic staining was not scored as positive (16).

### Western Blots

Equal concentration of total cell line extracts from UACC62, MOLT4, LOXIMVI, RXF393, and OVCAR5 or recombinant, e.coli produced protein encoding four different human proteins (AbNova Inc) were run on a denaturing polyacrylamide gel, transferred to blotting paper. Blots were exposed to either a monoclonal antibody (s0722) directed at a synthetic peptide encoded within TRMT2A amino terminal third of the protein, a polyclonal antibody directed at the same peptide (s0545), or an independent polyclonal affinity-purified antibody (s0728) directed at a distinct, non-overlapping peptide in the middle third of the protein.

### Discovery and validation of HER2 prognostic biomarker

We previously reported the screening of 126 novel and commercially available antibodies on the CCIH tissue array[16]. Prior to the analysis performed in this manuscript, thirty seven of these antibodies were selected for staining of the CCF tissue array cohort solely on the basis of a subjective assessment of staining quality and sub-classification of breast cancer cases in the CCIH cohort. In order to identify biomarkers associated with outcome in the CCIH cohort, Cox proportional hazard analysis was used to identify the subset of these thirty-seven markers that had a univariate association of IHC staining with recurrence-free interval (distant and local recurrence) at 5 years. Antibody directed at TRMT2A was identified as the single marker associated with outcome in *HER2*+ patients. The hypothesis that TRMT2A was related to outcome in *HER2*+ patients was subsequently evaluated in the existing CCF staining dataset as a prospectively designated hypothesis. The RPCI cohort was assembled explicitly to test the association identified and confirmed in the CCIH and CCF cohorts respectively. Staining was completed and scores were attached to the patient code number by the pathology group before the clinical records were linked to the immunohistochemistry dataset by the clinical investigator.

### Statistical Considerations

The relationship between all recurrence within five years of diagnosis and TRMT2A staining in *HER2*+ patients was assessed by a Cox proportional hazards model. Independence of TRMT2A from other clinical and pathologic prognostic parameters was also tested via Cox proportional hazards with each variable in a bi- or multivariable model. All univariate and multivariable Cox proportional hazard ratios and associated p-values were calculated using S-plus software (Tibco Software, Palo Alto, California). All reported p values are two sided.

## Results

### Discovery study

**Figure 1:**
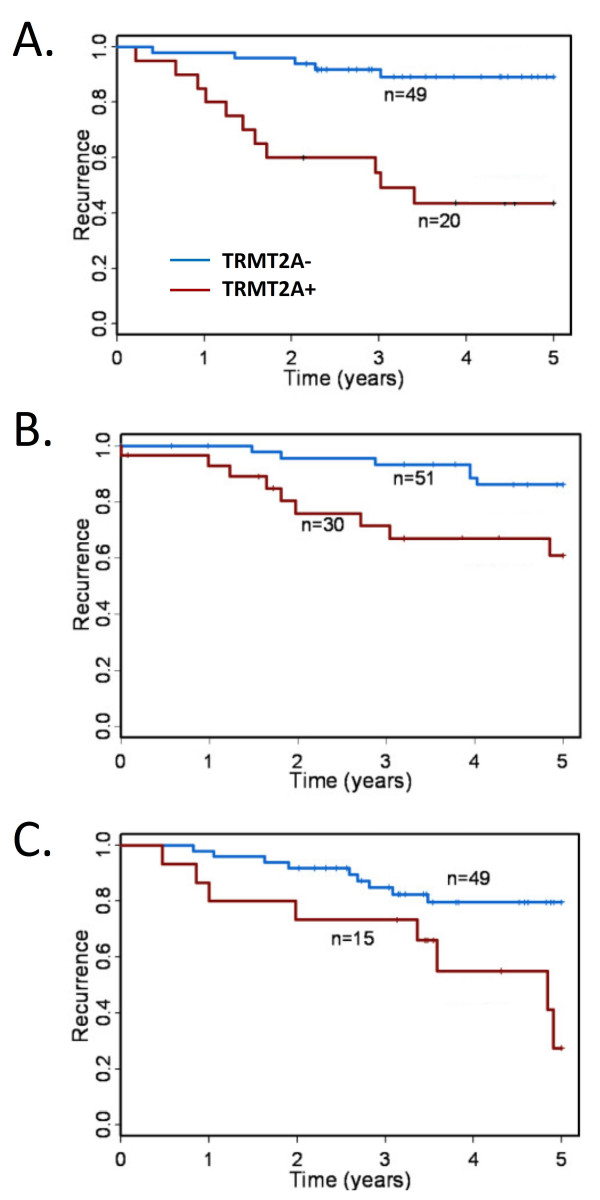
**Kaplan-Meier survival curves for HER2 positive breast cancer cases stratified by staining with antibody directed at TRMT2A**. TRMT2A expression is associated with outcome in a) the CCIH training cohort, b), the CCF confirmation cohort, and c) the RPCI validation cohort.

**Figure 2:**
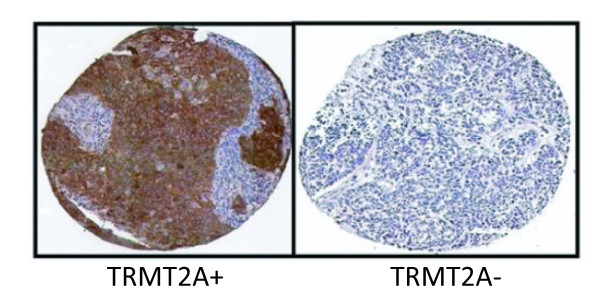
Immunohistochemical staining of HER2 + breast cancer with monoclonal anti-TRMT2A (S0722).

We previously described a breast cancer TMA assembled from archived tumor samples seen at a regional cancer referral center, the Clearview Cancer Institute of Huntsville (CCIH) that included 69 *HER2 *expressing cases. As part of a large project aimed at translating the emerging gene expression based classification of carcinoma into an IHC-based classification, we had screened a large number of novel polyclonal affinity-purified antibodies for association with clinical outcome and selected thirty-seven for further study in an independent cohort assembled at the Cleveland Clinic Foundation (CCF)[16]. Of these thirty-seven antibodies, only antibody targeting TRMT2A showed a strong correlation with likelihood of recurrence at five years in *HER2 *positive patients (HR 7.0; 95% CI 2.4 to 20.1, p < 0.0004) in the CCIH cohort (figure 1A). TRMT2A stained cases showed a strong uniform granular cytoplasmic staining of tumor cells (Figure 2). Occasional cases show some staining of nuclei, but only the presence of cytoplasmic staining was considered for the purpose of assessing staining with TRMT2A. Nodal status but not tumor size was associated with outcome in *HER2 *expressing patients in this cohort (Table 2).

**Table 2:** Clinical and pathologic prognostic factors in the clinical cohorts

	CCIH			CCF			RPCI		
	**Univariate HR**	**Factor Bivariate HR**	**TRMT2A Bivariate HR**	**Univariate HR**	**Factor Bivariate HR**	**TRMT2A Bivariate HR**	**Univariate HR**	**Factor Bivariate HR**	**TRMT2A Bivariate HR**

ER	**0.435 (0.11)**	**1.05 (0.93)**	**7.15 (0.0012)**	0.293 (0.014)	0.233 (0.044)	1.415 (0.63)	**0.471 (0.13)**	**0.573 (0.3)**	**2.573 (0.066)**

PR	**0.347 (0.16)**	**1.04 (0.97)**	**7.08 (0.0024)**	0.205 (0.036)	0.143 (0.066)	2.703 (0.082)	**0.18 (0.1)**	**0 (0.73)**	**2.8 (0.058)**

Age	**1.01 (0.52)**	**1.01 (0.61)**	**6.91 (0.00035)**	0.982 (0.29)	0.974 (0.15)	3.457 (0.02)	**1 (0.87)**	**1 (0.85)**	**3.39 (0.013)**

N Stage	**4.16 (0.00043)**	**3.9 (0.0027)**	**5.63 (0.0015)**	2.47 (0.0001)	2.41 (0.0002)	2.03 (0.23)	**1.17 (0.52)**	**1.37 (0.3)**	**3.33 (0.014)**

T Stage	**1.2 (0.54)**	**1.07 (0.85)**	**6.93 (0.00046)**	2.34 (0.0001)	2.72 (0.0001)	2.31 (0.13)	**1.24 (0.26)**	**1.14 (0.56)**	**3.35 (0.014)**

Grade	**0.869 (0.74)**	**0.805 (0.59)**	**12.25 (0.00018)**	2.16 (0.079)	2.35 (0.092)	2.79 (0.058)	**3.5 (0.018)**	**2.96 (0.077)**	**3.34 (0.017)**

Chemo	**1.64 (0.36)**	**0.925 (0.89)**	**7.14 (0.0005)**	8.78 (0.0048)	15.13 (0.0098)	2.23 (0.17)	**1.46 (0.42)**	**1 (0.99)**	**3.34 (0.014)**

Hazard ratios (p-values) of common prognostic factors within the Her2+ patients in each of the three cohorts. The first column reports each factor as univariate HR's whereas the second and third column reports each factor and TRMT2A respectively as bivariate HR's.

### Validation studies

We prospectively designated TRMT2A as a candidate biomarker for validation *in silico *in the existing CCF cohort dataset which confirmed the association between TRMT2A staining and poor outcome (HR 3.6; 95% CI 1.3 to 10.2, p < 0.02) (Figure 1B). We subsequently assessed data generated using all remaining thirty-six biomarkers and found that in addition to TRMT2A, both estrogen receptor and progesterone receptor expression were associated with outcome in this cohort. Neither pathologic or clinical stage were associated with outcome in this cohort.

**Figure 3:**
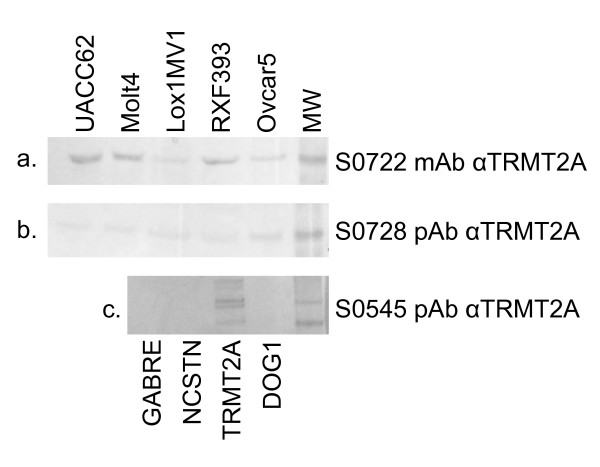
**Western blot staining with anti-TRMT2A antibodies**. a. Monoclonal antibody (S0722) directed at TRMT2A recognizes 38 Kd bands in five cell lines. b. Affinity purified polyclonal antibody (s0728) directed at a different, non-overlapping peptide in TRMT2A compared to the monoclonal also recognizes a 38 Kd band in the same cell lines. c. Polyclonal antibody to the same peptide as used to generate the monoclonal (s0545) stains only recombinant produced TRMT2A and not other recombinant proteins.

The identification of an association between TRMT2A expression and outcome in two independent cohorts encouraged us to explore the association in a novel third independent cohort assembled at the RPCI. Within this cohort, only grade was associated with outcome in *HER2 *expressing patients while estrogen receptor, progesterone receptor, nodal status, stage, and treatment with cytotoxic chemotherapy were not. We used a new rabbit monoclonal antibody directed at TRMT2A (S0722) which stained in a pattern very similar to the original polyclonal reagent across the CCIH cohort (data not shown). Western blot analysis with the rabbit monoclonal antibody showed an identical 38KD bands on multiple cell lines as the original polyclonal antibody and the same molecular weight band as a second polyclonal affinity purified antibody (s0728) directed at an independent epitope within the TRMT2A predicted protein. It also recognized only its cognate 68 kd in vitro transcribed and translated fusion protein in a western blot of four different fusion proteins loaded in equimolar amounts (figure 3). Cytoplasmic staining with the monoclonal reagent was highly associated with disease recurrence in the RPCI cohort (HR 3.42; 95%CI 1.3-8.9, p < 0.02) (Figure 1C).

In these three institutional cohorts from CCIH, CCF, and RPCI, TRMT2A stained 29%, 37% and 23% respectively. However, these subsets of patients contained 69%, 59% and 47% of the total number of *HER2*+ patients that recurred. Within the CCIH and CCF cohorts, where comparison with *HER2*- patients was possible, the five year recurrence rate of *HER2*+/TRMT2A-patients was lower than that of *HER2*- patients (CCIH:10% versus 19%, CCF: 12% versus 17%).

To assess whether TRMT2A was providing information in HER2 expressing patients that was independent of standard clinical and pathologic factors in addition to ER and PR staining, we performed univariate and bivariate analysis with each prognostic factor and outcome (Table 2). While TRMT2A was independent of each of these factors in the CCIH and RPCI cohorts, it was only independent of grade and age in the CCF cohort where the standard markers showed a much stronger univariate association with outcome. TRMT2A was independent or near independent of ER and PR in all three cohorts with the exception of ER in the CCF cohort. By multivariable analysis TRMT2A was independent of tumor size, nodal status and grade in both the CCIH and RPCI cohorts (Table 3).

**Table 3:** Mutivariable analysis

	CCIH		CCF		RPCI	
	**HR**	**p**	**HR**	**p**	**HR**	**p**

TRMT2A	7.78	0.0046	1.24	0.75	2.958	0.036

T stage	1.06	0.9	2.19	0.012	0.832	0.51

N stage	4.15	0.023	1.44	0.26	1.517	0.27

Grade	0.85	0.7	2	0.24	3.205	0.062

		n = 57		n = 70		n = 59

## Discussion

In the current report, we have demonstrated in three independent institutional cohorts that IHC-based detection of cytoplasmic TRMT2A identifies a subset of *HER2*+ patients with a high likelihood of recurrence. In these cohorts in which patients were treated with various cytotoxic chemotherapy regimens without Traztuzumab, estrogen and progesterone receptor expression showed an inconsistent relationship with outcome similar to inconsistent finding in the published literature [13-15]. TRMT2A's association with outcome in bivariable analysis was independent or near independent of both pathologic grade and hormone receptor status. These findings suggest that TRMT2A is a novel, independent biologic factor expressed in tumors associated with clinical outcome in HER2 expressing breast cancer. Although the association between TRMT2A expression and outcome was independent of chemotherapy treatment, it should be noted that as these cohorts were not derived from randomized trials it remains unclear whether TRMT2A has primarily a prognostic relationship to outcome or if it is predictive of response to therapy.

TRMT2A was chosen for study based upon its identification as a novel open reading frame whose expression varies during the cell cycle similar to the oscillation of MCM, tubulin, CDC2, and other genes whose expression peak in S-phase and decrease during mitosis. Genes with similar expression patterns have been noted to be involved in different aspects of transiting the cell cycle and certain members have been noted to be elevated in tumor cells[17,18]. The function of TRMT2A, a 676 amino acid protein, is not known although by sequence homology it has a domain related to RNA methyltransferases[19]. Its expression has been previously shown to correlate with poor prognosis in ER+ breast cancer as a member of a validated, multivariate index assay combining five IHC antibodies in a prognostic test for Tamoxifen treated ER+ breast cancer [16,20]. When present in tumor cases, TRMT2A protein expression typically demonstrates a homogenous cytoplasmic pattern with some additional staining of nuclei. Nuclear staining alone is not associated with outcome (data not shown) and is ignored for the purpose of pathologic evaluation. This raises the possibility that cytoplasmic expression reflects a tumor specific aberrant expression pattern. TRMT2A does not stain intermittent cycling cells in a manner reminiscent of classic makers of proliferation (e.g. KI67/Mib1 and PCNA). KI67/Mib1 staining was not significantly prognostic is any of three her2 cohorts reported herein although its expression trended towards an association with outcome (data not shown). Therefore, TRMT2A appears to be measuring physiology distinct from that measured by classic proliferation markers. Given TRMT2A's strong prognostic association in HER2 expressing patients and its periodic expression during the cell cycle, it is interesting to speculate that TRMT2A over-expression may reflect tumor cells driven by an activated HER2 pathway towards aggressive growth.

## Conclusion

A greater understanding of the biologic heterogeneity within the HER2 positive cohort of breast cancers identified clinically would aid efforts to assess and predict outcome for this important subset of patients. Furthermore, prudent selection of treatment regimen and length of treatment could be helped by the identification of biomarkers that identify patients with a more aggressive clinical course as well as those most likely to respond to adjuvant therapy. Our data suggests that *HER2*+/TRMT2A+ breast cancers are more likely to recur treated with traditional cytotoxic therapy alone and therefore more aggressive treatment in the adjuvant setting may need to be considered for these patients, regardless of the other conventional risk factors. Whether or not *HER2*+/TRMT2A+ tumors are more likely to show benefit from trastuzumab cannot be determined from the current data but these data suggest that it should be studied in the clinical trial setting to further explore its relationship to response to adjuvant cytotoxic chemotherapy and *HER2 *targeted therapy.

## Competing interests

David G. Hicks, Bagi R. Janarthanan, R Vardarajan, Swati Kulkarni, Thaer Khoury, Daniel Dim, G. Thomas Budd, Brian J. Yoder, Raymond Tubbs, report no conflict of interest.  Rodney A. Beck, Brian Z. Ring, Robert S. Seitz, Douglas T. Ross are employees of, and stockholders in Clarient Inc which funded studies reported in this manuscript.  Marshall T. Schreeder, Noel C. Estopinal are stock holder of Clarient Inc which funded studies reported in this manuscript.

## Authors' contributions

DGH, BRJ, RV, SK, TK, DD, GTB, BJY, RT, MTS and NCE assembled paraffin blocks and clinical follow-up information for the cohorts reported in this manuscript. RAB performed wet work and IHC evaluation, BZR, RSS, performed the statistical analysis. DTR, BZR, RSS and DGH conceived the study and wrote the manuscript. All authors approved the final manuscript.

## Pre-publication history

The pre-publication history for this paper can be accessed here:

http://www.biomedcentral.com/1471-2407/10/108/prepub
